# Mapping Knowledge Structure and Global Research Trends in Gout: A Bibliometric Analysis From 2001 to 2021

**DOI:** 10.3389/fpubh.2022.924676

**Published:** 2022-06-29

**Authors:** Pengfei Wen, Pan Luo, Binfei Zhang, Yumin Zhang

**Affiliations:** Department of Joint Surgery, Honghui Hospital, Xi'an Jiaotong University, Xi'an, China

**Keywords:** gout, gouty arthritis, knowledge structure, bibliometric analysis, hotspots, research trends

## Abstract

**Background:**

The incidence and prevalence of gout have been steadily increasing globally, which has resulted in gout research attracting consistently increased attention. This study aimed to visualize the knowledge structure and research trends in gout research through bibliometrics to help understand the future development of basic and clinical research.

**Methods:**

Articles and reviews on gout from 2001 to 2021 were extracted from the Web of Science Core Collection database. CiteSpace and VOSviewer software were used to visualize the knowledge network of countries, institutions, authors, references, and keywords in this field. SPSS and Microsoft Excel software were used for curve fitting and correlation analysis.

**Results:**

A total of 3,259 articles and reviews were included. The number of publications about gout significantly increased yearly. Publications were mainly concentrated in North America, Europe, Oceania, and East Asia. The USA contributed most with 1,025 publications, followed by China and New Zealand. After adjusting for publications by population size and Gross Domestic Product (GDP), New Zealand ranked in the first place. GDP and international collaboration were significantly correlated with scientific productivity for gout research. University of Auckland and Professor Dalbeth Nicola were the most prolific institutions and influential authors, respectively. Rheumatology was the most productive journal for gout research. Gout research hotspots have shifted over time in the following order: clinical features, pathological mechanisms, complications, gouty arthritis, epidemiology, and dual-energy computed tomography to drug clinical trials, which can be observed from the keyword analysis and co-cited reference cluster analysis.

**Conclusions:**

This study found that research on gout is flourishing. The development and experimentation of drugs for the prevention and treatment of gouty arthritis would be the focus of current research and developmental trends in future research.

## Introduction

Gout is the most common inflammatory arthritis worldwide, with an incidence of 0.6 to 2.9 per 1,000 people-years and a prevalence ranging from <1 to 6.8% reported in population-based studies (1, 2). Furthermore, these epidemiological data seem to be steadily increasing globally (3). Gout results from persistently elevated serum uric acid levels (hyperuricaemia) which leads to the deposition of monosodium urate (MSU) crystals in joints, tendons and other tissues, triggering recurrent episodes of apparent acute inflammation known as gout flares. Many factors contributing to hyperuricaemia are also risk factors for gout incidence, including genetic, age, gender, and social and economic factors (4, 5); greater consumption of purine-rich foods such as red meat, seafood, alcohol, and sugary beverages (6, 7); and multiple other metabolic syndromes such as hypertension, abnormal lipid/glucose metabolism, and chronic kidney disease (7). Although gout is a curable rheumatic disease, many patients do not receive regular urate-lowering therapy (ULT) or inadequate management, which leads to advanced gout characterized by tophi, chronic gouty synovitis and structural joint damage (2, 8). Gout is also commonly associated with other conditions such as hypertension, obesity, cardiovascular disease, diabetes mellitus, chronic kidney disease, and kidney stones (9).

Given the above aspects, gout has received increasing attention from scholars, and a large number of studies on gout have been published. The rapidly growing number of publications makes it increasingly difficult for researchers to fully understand, evaluate and identify the most relevant and valuable information in the field, especially for new investigators. Although some systematic reviews and meta-analyses surrounding gout could provide researchers with innovations and basic information, these summative reviews focus only on a unique perspective of gout research without offering a comprehensive overview of macroscopic information about the field, such as numerical growth trends, contributions of countries, institutions, and authors, and the evolution of research themes. For example, Choi et al. (10) reviewed the key aspects of gout pathogenesis, while Dehlin et al. (7) focused on the epidemiology of gout.

Bibliometrics, a feasible method, can quantitatively and qualitatively analyze the scientific achievements, provide an objective and holistic overview, discover the spatial and temporal distribution of research status, identify high-impact articles, authors, and institutions, reveal research hotspots and trends, and ultimately contribute to the advancement of the research field (11). It has been widely used in medical fields such as orthopedics (12), COVID-19 (13), oncology (14), and rheumatology (15). Zhang et al. (16) conducted bibliometrics research to discuss publication status and research hotspots in hyperuricaemia. In 2013, Gerber et al. (17) performed the first bibliometric analysis for publications on gout during the period 1900 to 2012 and identified the most prolific authors, institutions, countries and journals in this field. However, this study did not analyze the keywords and co-cited references to reveal research hotspots. In addition, with the advancement of technology, studies on gout have sprung up over the last decade. Hence, this study aimed to comprehensively analyze the scientific publications on gout research from 2001 to 2021, so as to determine the current research status and knowledge structure and to predict the future development prospects.

## Materials and Methods

### Data Sources and Search Strategies

**Figure 1:**
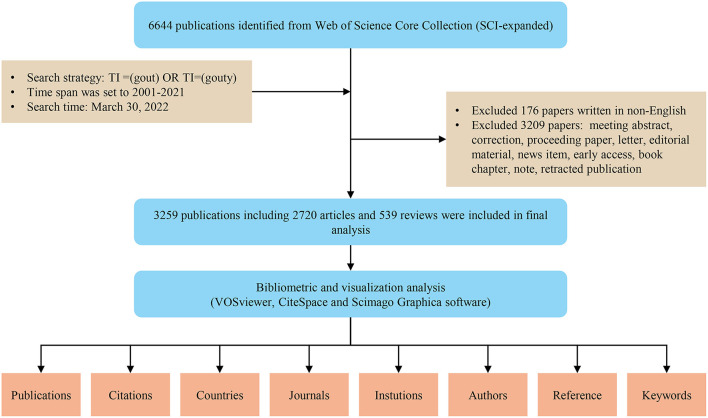
Flowchart of the literature search, screening, and analysis.

Web of Science (WoS) contains more than 12,000 international academic journals, which is one of the most comprehensive and authoritative database platforms for accessing global academic information and is widely used for bibliometric analysis (15, 18). WoS Core Collection (WoSCC) database is a typical citation database containing literature abstracts and other relevant data such as citation and research collaboration information, facilitating bibliometric analysis (19). Moreover, it could directly provide reference files that met the specific format requirements as dictated by bibliometric software (11, 15). In our study, all literature was retrieved and downloaded from the Science Citation Index Expanded of WoSCC database on March 30, 2022, to avoid bias caused by database updates. Since gout was the main subject of this study, a search was performed for terms related to gout according to the title (TI) to obtain more precise results. The specific search strategy was as follows: TI = (gout) OR TI = (gouty), and the time span was set to 2001–2021. The term gouty was to retrieve gouty arthritis, gouty inflammation, gouty tophus/tophi, gouty Arthropathy, gouty patients/population, gouty nephropathy, and so on. A total of 6,644 publications were retrieved, of which 3,385 invalid records were excluded, including essays, edited materials, corrections, meeting summaries, letters, early visits, retracted publications and non-English literature works by referring to previous literature (15, 18, 20). Finally, 3,259 valid publications were obtained as the final dataset for further analysis (Figure 1).

### Data Extraction

Data extraction was performed by two independent researchers to ensure the accuracy and reliability of the results. Any disagreements between the two researchers were discussed until a consensus was reached. The extracted data included authors, titles, publication years, citation times, countries, institutions, journals, highly cited articles, references, and keywords. The Hirsch index (H-index) was obtained by WoS. Journal information including impact factor (IF) and quartile in category (Q1–Q4) were collected from the 2021 Journal Citation Report (12). In addition, taking into account the differences in economic and demographic conditions in different countries, several ratio indexes were introduced, including the number of papers per million people and the number of papers per trillion Gross Domestic Product (GDP) (15).

### Data Analysis

Statistical analysis was performed using IMB SPSS 22.0 (IBM Corp., Armonk, NY, USA), and Microsoft Excel 2016 (Microsoft Corp., Redmond, WA, USA). Statistical plots were drawn using OriginPro 9.1 (OriginLab Corp., Northampton, Massachusetts, USA). Categorical data were expressed as counts (percentages). The correlation strength between continuous variables was assessed by Spearman's correlation coefficient. *P* < 0.05 was defined as statistically significant.

Visualization software including VOSviewer, CiteSpace and Scimago Graphica were used in the bibliometrics analysis. Scimago Graphica software was used to map the global distribution of national publications and the cooperation network of countries. VOSviewer software was used to visualize the co-authorship analyses of institutions and authors with a document threshold of 15, as well as the co-occurrence analysis of keywords with an occurrence threshold of 5. CiteSpace was mainly used for reference co-citation analysis, keyword burst detection, and drawing timeline views of reference clusters. In the visual graphs, the colors of the nodes and connections represent the period in which the articles were published. The overall time span from 2001 to 2021, with 3 years per slice, was divided into 7 different time slices corresponding to 7 different colors.

## Results

### Trends in Publications and Citations

**Figure 2:**
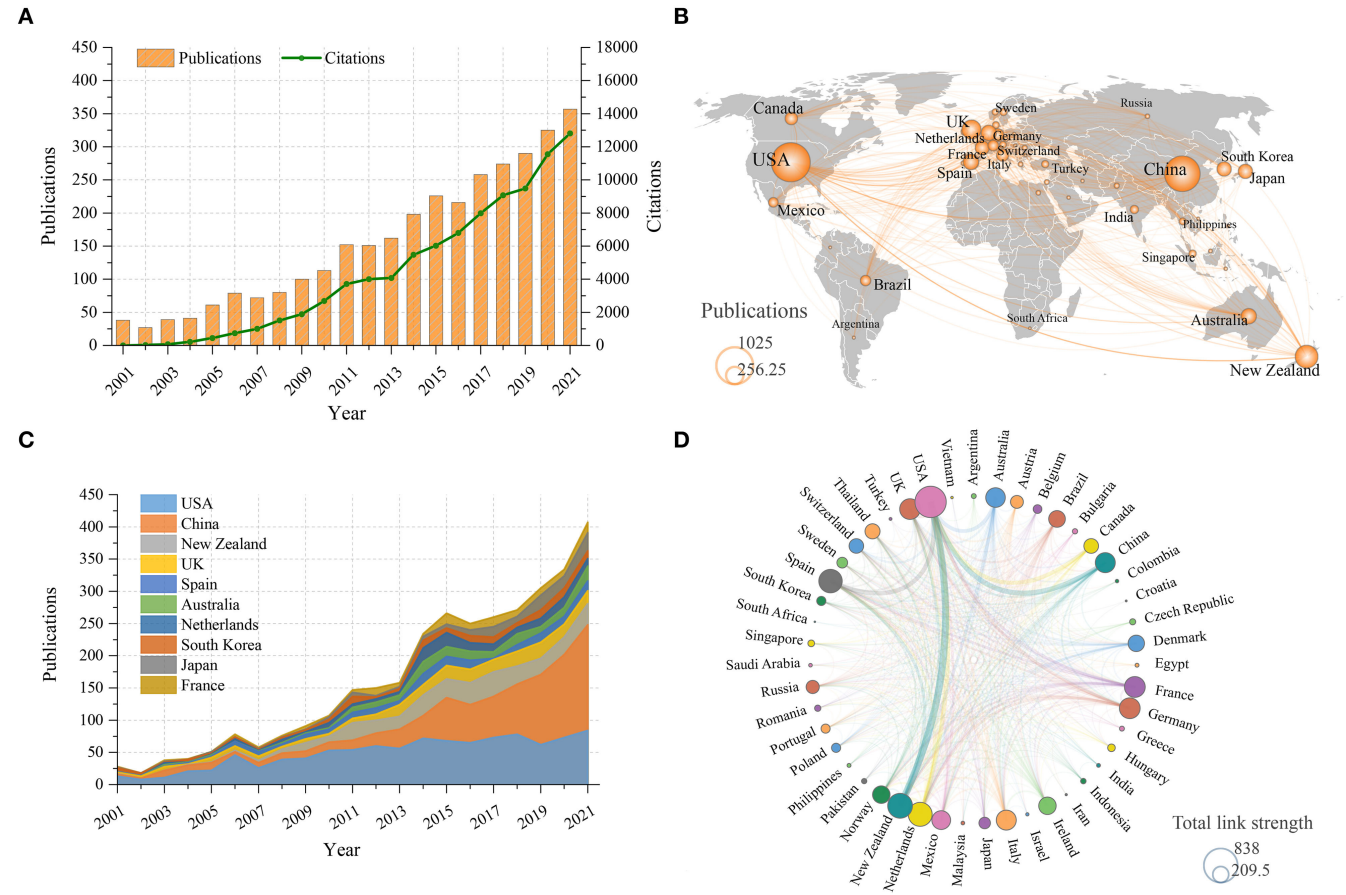
**(A)** Trends in annual citations and publications about gout from 2001 to 2021. **(B)** World map showing the contribution of each country. **(C)** Trends in annual publication count in the top 10 productive countries. **(D)** Cooperation network among countries. The thickness of lines between two countries indicates the strength of cooperation.

After the literature screening, a total of 3,259 publications were included in the final analysis, including 2,720 original articles and 539 reviews. The detailed distribution of annual publications for gout research was shown in Figure 2A. It can be seen that the number of annual publications on gout showed an overall upward trend and reached its peak in 2021 with 357 articles. When it comes to the number of citations, the cumulative total number of citations for these publications was 92,016 (51,357 after removing self-citations), the average number of citations per publication was 28.23 and the H-index was 130. As can be seen from the distribution of annual citations (Figure 2A), it showed a linear growth trend (R^2^ = 0.9289).

### Global Research Status and Knowledge Structure

#### Analysis of Countries

From the world map of country contributions (Figure 2B), it can be observed that the vast majority of works were published by researchers from North America, Europe Oceania, and East Asia. Specifically, as shown in Table 1, the USA has published the most papers in this field, with 1,025 (31.5%) papers, followed by China, New Zealand and the United Kingdom (UK), while other countries have published <200 papers. Moreover, after adjusting for publications by population size and GDP, New Zealand occupied the first position with 1,661.12 papers per trillion GDP and 6,883.94 papers per million people. In addition, the study found there was a high positive correlation between the number of publications and GDP (*r* = 0.826, *p* < 0.001) (Supplementary Table 1).

**Table 1:** Top 10 countries with the most publications.

**Rank**	**Country**	**No. of papers**	**Total citation**	**Average citation**	**H-index**	**No. of papers per trillion GDP**	**No. of papers per million people**
1	USA	1,025	43,438	42.38	101	48.92	311.09
2	China	854	10,799	12.65	41	58.01	60.53
3	New Zealand	350	12,093	34.55	54	1,661.12	6,883.94
4	UK	245	11,872	48.46	52	88.77	364.50
5	Spain	162	9,623	59.40	47	126.42	342.12
6	Australia	160	3,821	23.88	34	120.50	622.88
7	Netherlands	158	7,773	49.20	38	172.89	905.90
8	South Korea	140	1,510	10.79	21	85.48	270.37
9	Japan	139	3,824	27.51	31	27.48	110.46
10	France	134	9,045	67.50	40	50.94	198.84

*GDP, Gross Domestic Product; The Demographic and GDP data were obtained from the World Bank official website (https://data.worldbank.org.cn/)*.

As shown in Figure 2C, the USA dominated the number of papers in this field until 2018, while China experienced rapid growth since 2012 and even surpassed the USA after 2018. Figure 2D showed the international cooperation among the different countries. The thickness of lines between two countries indicates the strength of cooperation. It can be seen that the USA had the closest cooperation with New Zealand, China, Netherlands and Spain. Furthermore, this study showed that the number of international collaborations were significantly correlation with the total publications (*r* = 0.865, *p* < 0.001), total citations (*r* = 0.864, *p* < 0.001), and average citations (*r* = 0.585, *p* < 0.001) (Supplementary Table 1).

#### Analysis of Institutions

**Figure 3:**
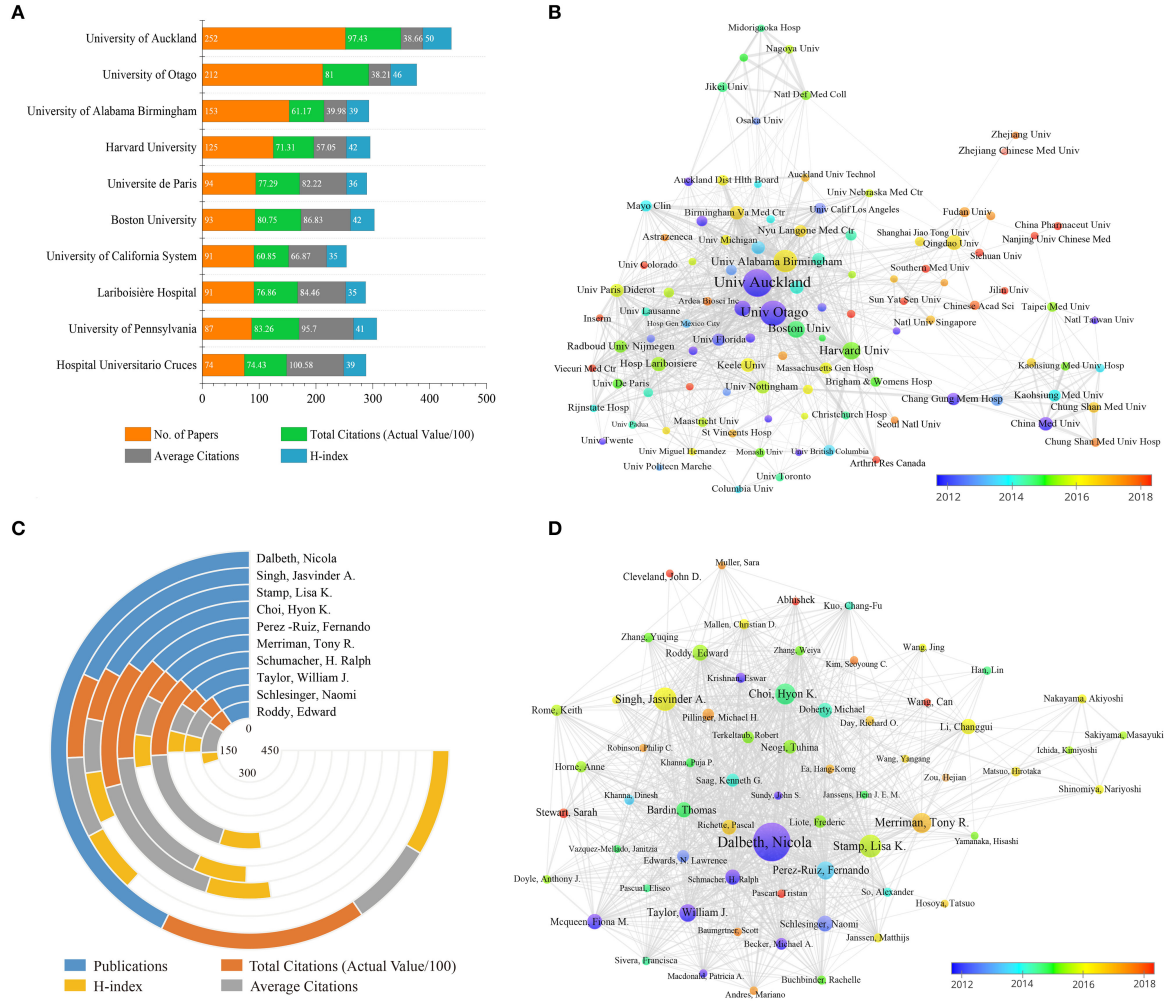
**(A)** The publication counts, citations and H-index of the top 10 prolific institutions. **(B)** Cooperation network among institutions. **(C)** The publication counts and citations of the top 10 most productive authors. **(D)** Cooperation network among authors. The nodes in the graph represent institutions or authors, and lines between the nodes represent the collaborative relationships. Nodes are marked with different colors depending on the average appearing year. The thickness of lines between two institutions or authors indicates the strength of cooperation, while the area of nodes represents the number of publications.

It was roughly estimated that more than 2,500 institutions contributed to this field. Figure 3A showed the top 10 most productive institutions. Five of these are from the USA, two from New Zealand, two from France, and one from Spain. Specifically, University of Auckland in New Zealand ranked first with 252 articles and H-index of 50, followed by University of Otago. Hospital Universitario Cruces in Spain had the highest average citation of 100.58.

Figure 3B highlighted the close and complex cooperation among different institutions. It can be seen that inter-institutional collaborations were scattered within high-income countries such as Oceanian, North American, and European countries. The cooperations centered on University of Auckland, University of Otago, and University of Alabama at Birmingham were the most frequent, indicating a strong influence of these institutions. In addition, several institutions marked in red, such as Viecuri Medical Center, University of Colorado, Arthritis Research Canada, Sichuan University, China Pharmaceutical University, Jilin University, Southern Medical University, and Sun Yat Sen University, were relatively new participants in the gout research.

#### Analysis of Journals

During the last two decades, a total of 499 academic journals published papers on gout. Table 2 summarized the characteristics of the top 10 most prolific journals. Of these, Rheumatology (159, 4.88) published the most, followed by Journal of Rheumatology (143, 4.39%) and Annals of the Rheumatic Diseases (123, 3.77%). Three of the top 10 academic journals belong to Q1, with Annals of the Rheumatic Diseases having the highest IF (19.103), followed by Arthritis and Rheumatology (10.995) and Rheumatology (7.58). As far as the research fields of these journals were concerned, 80% of these journals were classified as rheumatology. In terms of publishers, four of these journals were from the UK, four from the USA, and the others from Canada and Germany, respectively. Notably, most of these active journals were located in Europe and North America.

**Table 2:** The top 10 journals contributing to publications.

**Rank**	**Journal**	**No. of papers (%)**	**Impact factor (2021)**	**Quartile in category**
1	Rheumatology	159 (4.88)	7.58	Q1
2	Journal of Rheumatology	143 (4.39)	4.666	Q2
3	Annals of the Rheumatic Diseases	123 (3.77)	19.103	Q1
4	Clinical Rheumatology	118 (3.62)	2.98	Q3
5	Arthritis Research and Therapy	114 (3.50)	5.156	Q2
6	JCR-Journal of Clinical Rheumatology	91 (2.79)	3.517	Q3
7	Arthritis and Rheumatology^†^	78 (2.39)	10.995	Q1
8	Rheumatology International	70 (2.15)	2.631	Q4
9	Arthritis Care and Research	67 (2.06)	4.794	Q2
10	International Journal of Rheumatic Diseases	60 (1.84)	2.454	Q4

† *Arthritis and Rheumatism relaunched as Arthritis and Rheumatology after 2015. The data from these journals were merged*.

### Overview of Landmark Articles and Authors

#### Analysis of Authors

In terms of the top 10 prolific authors (Figure 3C, Table 3), Dalbeth Nicola from University of Auckland ranked first, followed by Singh Jasvinder A. and Stamp Lisa K. In addition, Dalbeth Nicola was also the top author with the highest H-index. Stamp Lisa K, Merriman Tony R, and Taylor William J. were from the same institution, University of Otago. Of note, four of the top 10 authors were from the USA institutions and four from Spain institutions. A visualization of the author's co-authorship analysis was generated by VOSviewer software (Figure 3D). It can be seen that Dalbeth Nicola had the most collaborations centered on him, indicating his strong influence. Several authors marked in red, such as Stewart Sarah, Abhishek, Wang Can and Cleveland John D, were new active participants in gout research.

**Table 3:** Top 10 most prolific authors on gout research.

**Rank**	**Author**	**No. of papers**	**Total citations**	**Average citations**	**H-index**	**Institution**	**Country**
1	Dalbeth, Nicola	247	9,741	39.44	50	University of Auckland	New Zealand
2	Singh, Jasvinder A.	105	4,343	41.36	32	UBA	USA
3	Stamp, Lisa K.	97	2,974	30.66	29	University of Otago	New Zealand
4	Choi, Hyon K.	87	8,125	93.39	41	MGH	USA
5	Perez-Ruiz, Fernando	76	7,459	98.14	38	Hospital Universitario Cruces	Spain
6	Merriman, Tony R.	75	2,437	32.49	27	University of Otago	New Zealand
7	Schumacher, H. Ralph	71	7,860	110.70	40	University of Pennsylvania	USA
8	Taylor, William J.	67	2,211	33.00	25	University of Otago	New Zealand
9	Schlesinger, Naomi	56	2,370	42.32	26	UMDNJ	USA
10	Roddy, Edward	55	3,344	60.80	23	Keele University	UK

*UBA, University of Alabama at Birmingham; MGH, Massachusetts General Hospital; UMDNJ, University of Medicine and Dentistry of New Jersey*.

#### Analysis of Highly Cited Literature

**Figure 4:**
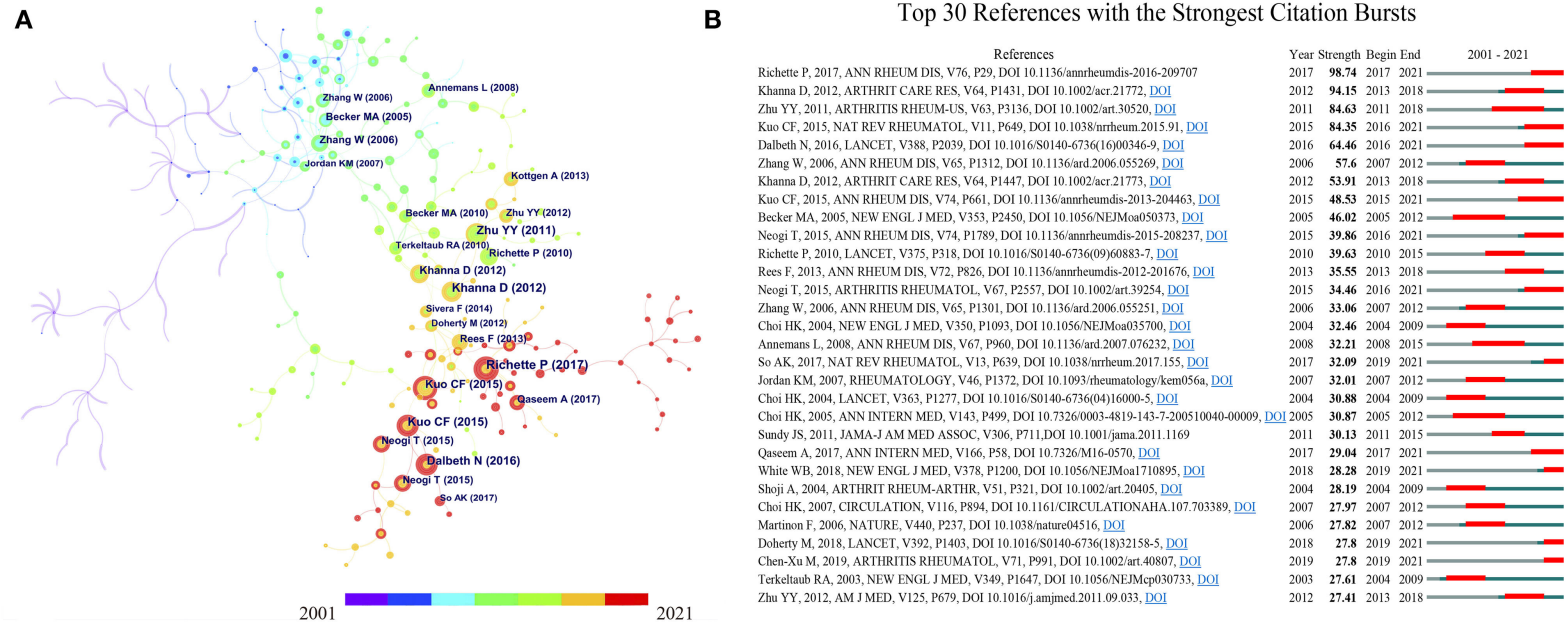
**(A)** The co-cited reference knowledge map. **(B)** The top 30 references with the strongest citation bursts.

Table 4 showed the details of the top 10 most cited papers on gout with citations ranging from 583 to 3,319. Seven of these articles were original articles and three were systematic reviews. Two of these articles were published in the New England Journal of Medicine (IF = 91.245) and one in the Lancet (IF = 79.321). One article published in Nature (IF = 49.962) was ranked first with 3,319 citations (21). Figure 4A illustrated a knowledge map of highly co-cited references. Figure 4B specifically showed the top 30 references with the strongest citation bursts, in which the blue line represented the time interval, and the red part indicated the period when the reference burst occurred. Among these references, the reference with the strongest citation burst value was written by Richette P et al. (24).

**Table 4:** Top 10 high-cited articles on gout research.

**Authors**	**Year**	**Article Title**	**Journal**	**Total citations**
Martinon F, et al. (21)	2006	Gout-associated uric acid crystals activate the NALP3 inflammasome	Nature	3,319
Zhu YY, et al.	2011	Prevalence of gout and Hyperuricemia in the US general population the national health and nutrition examination survey 2007–2008	Arthritis and Rheumatism^†^	1,084
Khanna D, et al. (22)	2012	2012 American College of Rheumatology guidelines for management of gout. Part 1: systematic nonpharmacologic and pharmacologic therapeutic approaches to hyperuricemia	Arthritis Care and Research	1,027
Becker MA, et al.	2005	Febuxostat compared with allopurinol in patients with hyperuricemia and gout	New England Journal of Medicine	824
Zhang W, et al. (23)	2006	EULAR evidence based recommendations for gout. Part II: management. Report of a task force of the EULAR standing committee for international clinical studies including therapeutics (ESCISIT)	Annals of the Rheumatic Diseases	810
Richette P, et al. (24)	2017	2016 updated EULAR evidence-based recommendations for the management of gout	Annals of the Rheumatic Diseases	656
Choi HK, et al. (10)	2005	Pathogenesis of gout	Annals of Internal Medicine	635
Choi HK, et al.	2004	Purine-rich foods, dairy and protein intake, and the risk of gout in men	New England Journal of Medicine	623
Richette P, et al.	2010	Gout	Lancet	594
Kuo CF, et al. (6)	2015	Global epidemiology of gout: prevalence, incidence and risk factors	Nature Reviews Rheumatology	583

† *Arthritis and Rheumatism relaunched as Arthritis and Rheumatology after 2015*.

### Overview of Research Hotspots and Frontiers

#### Cluster Analysis of Co-cited References

**Figure 5:**
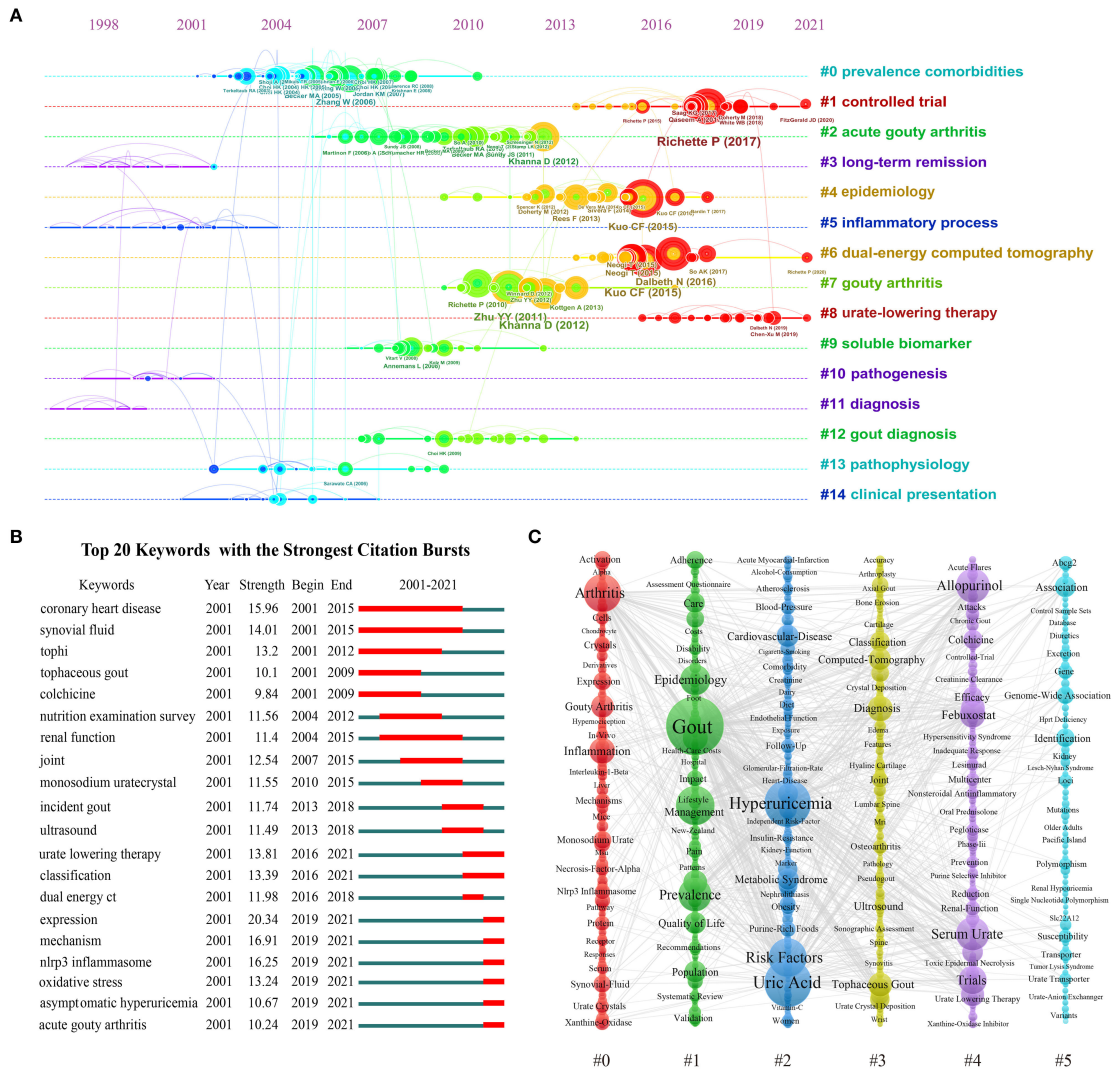
**(A)** The timeline graph of co-cited reference clusters. **(B)** The top 20 keywords with the strongest citation bursts. **(C)** The co-occurrence view of the keywords, in which each column represents a cluster of studies generated by VOSviewer.

The references in the co-cited network (Figure 4A) were divided into 15 different clusters by cluster analysis shown in the timeline view (Figure 5A). These clusters were labeled by extricating terms from the titles of cited publications using the log-likelihood ratio algorithm due to its better performance in covering the “uniqueness and coverage” of all labels (11, 18). As can be seen in Figure 5A, the cluster of prevalence comorbidities was the largest (#0), followed by controlled trial (#1) and acute gouty arthritis (#2). Moreover, the evolution characteristics of each cluster can be known from this timeline view. As one can see, the clusters of controlled trials (#1), dual-energy computed tomography (#6), and ULT (#8) have been the hot topics of gout research in recent years and still continue to be.

#### Analysis of Keywords

In the keyword analysis, the meaningless keywords were excluded and the same meaning keywords were merged. Figure 5B listed the top 20 keywords with the strongest citation burst, and the keywords that the burst period lasted until now were ULT, expression, mechanism, nlrp3 inflammasome, oxidative stress, asymptomatic hyperuricemia, and acute gouty arthritis. Figure 5C showed a visualization of keywords that co-occurred more than 5 times in the gout research. The size of the node was proportional to the frequency of keyword occurrences, and a thicker line between two nodes was associated with a higher frequency of their co-occurrence (14, 15). A total of 846 keywords were divided into 6 groups. According to the keywords in the groups, the groups can be summarized as follows: #0 gouty arthritis, #1 epidemiology, #2 complications and risk factors, #3 diagnostics, #4 treatment, #5 genetics, and urate metabolism.

## Discussion

This study provided a bibliometric analysis of gout research from 2001 to 2021 to find milestone achievements and predict new research hotspots. It can also help beginners intuitively and systematically understand the development process and trends in this field. Zhang et al. demonstrated the publication status of hyperuricaemia through bibliometric analysis of 6,313 articles based on the WoS database and predicted that “pathophysiology,” “hyperuricaemia and cardiovascular disease”, “hyperuricaemia and gout,” and “genome-wide research” would be the next hotspots (16). However, the researchers did not perform a collaborative analysis and focused on hyperuricaemia rather than gout. Although hyperuricaemia is a major risk factor for gout, most patients with hyperuricaemia are asymptomatic and do not develop gout (3). Even in patients with severe hyperuricaemia (higher or equal to 0.60 mmol/L; 10mg/dL), less than half of them develop gout within 15 years (25). Gerber et al. conducted a bibliometric analysis of studies on gout from 1900 to 2012 and identified the most prolific authors, institutions, countries and journals, but did not analyze the research hotspots (17). There are some strengths of this study. The combination of knowledge maps and visual analysis could simultaneously reflect the current status of research quantitatively and show the distribution of collaboration among countries, institutions and authors. Secondly, this study reported the factors that facilitate national scientific output. Finally, we showed the evolution of research hotspots clearly through co-cited reference analysis and keyword analysis.

This study found that the number of publications and citations of gout have been numerous and increasing in the past two decades (Figure 2A). Accordingly, we believe that gout has attracted a lot and growing attention from scholars, and it is an important research field all over the world. This trend may be related to the increasing incidence and prevalence of gout worldwide (2, 7), probably due to inadequate management and poor adherence of gout patients, as well as to the changing age structure of the world population (development of aging) since studies have demonstrated that the prevalence of gout increases with age (7, 26).

From the perspective of national contribution, the USA ranked first in the world in terms of publications, citations and H-index, indicating its dominant influence (Figure 2B, Table 1). After adjusting the number of publications by population size and GDP, New Zealand was in the first place and significantly more than other countries, followed by the Netherlands and Australia (Table 1). The influence and contribution of scientific institutions fundamentally represent a country or region. The study showed that two of the top 10 prolific institutions were located in New Zealand, five in the USA, two in France, and one in Spain (Figure 3A). In summary, most of the works were produced in high-income countries, such as those in North America, Oceania, Europe, and East Asia. This study found a significant positive correlation between country output and GDP, indicating that economic power was an important factor affecting scientific activities. Countries with high GDP may allot substantial investments in scientific investigations and foster a large number of senior researchers (15, 27). However, the lack of studies in developing nations may misrepresent and underestimate the actual burden of gout in these countries. From the perspective of gout epidemiology, the regional imbalance in the article output may be attributed to the highest prevalence of gout in Oceania globally, particularly in indigenous and South Pacific island populations, for which a prevalence exceeding 10% has been reported. Studies from Australia reported a prevalence of gout in adults over 20 years old ranging from 1.5 to 6.8%. Furthermore, studies from Europe and North America also reported a higher prevalence of gout (1, 2, 28), while developing countries had the lowest prevalence (29). Of note, although China ranked second in terms of the total number of papers (Table 1) and dominated after 2018 (Figure 2C), transnational collaboration and information exchange between China and other countries were scarce (Figures 2D, 3B). This study suggested that the number of international research collaborations is a strong factor facilitating the productivity and impact in gout research. The results of this study agree with previous studies that have shown that countries with more international collaborations tend to publish articles with greater scientific impact compared to those with less open collaborations (30). Therefore, it is strongly recommended that Chinese institutions would improve international collaborations and exchanges to promote gout research and development.

Publishing research results in international peer-reviewed journals is an important part of establishing effective scientific communication (15). The analysis of the distribution of journal sources could help researchers quickly find the most suitable journals for their works. In this study, Annals of the Rheumatic Diseases had the highest IF among the top 10 prolific journals, followed by Arthritis and Rheumatology, representing their high reputation and authority in this field. Notably, although East Asia was also one of the major contributors to this field, there were very few influential journals, which suggested that Asian countries should strengthen the development of international journals to further enhance their academic influence in this field.

The number of scientific articles published by an author can be a good representation of his research activities and contributions to the field. The data from Dalbeth Nicola (University of Auckland, New Zealand) were particularly attractive in terms of publications and citations (Table 3, Figure 3C). He has contributed to 247 publications on gout research with a total of 9,741 citations. In addition, the largest number of collaborations centered on Dalbeth Nicola indicates his strong academic influence on gout research (Figure 3D). By analyzing Table 4 and Figure 4, it can be found that the top 10 cited publications (Table 4) were all included in the references with the strongest citation bursts (Figure 4). In other words, literature with high citations can be considered the most valuable and influential research in the field, so new researchers can read these papers before conducting further research. For example, the most highly cited study published in Nature showed that MSU engaged the caspase-1-activating NALP3 inflammasome, resulting in the production of active interleukin (IL)-1beta and IL-18, which had a profound impact on the study of gout flare mechanism and targeted therapy (21). The guideline for the management of gout recommended by the American College of Rheumatology provides direction for clinicians and patients making decisions on the management of gout and was updated in 2020 (22, 31). Additionally, the European League Against Rheumatism (EULAR) taskforce introduced recommendations for the management of gout based on expert opinions (23). This recommendation was subsequently updated based on extensive new research evidence and quickly gained a large number of citations and wide acceptance by researchers (24). This article also had the strongest citation burst of 98.74, and its burst period has lasted until now (Figure 4B).

Cluster analysis of co-cited references and co-occurrence keywords has been proved to reflect the research topics and hotspots in the field (32, 33). In this study, co-cited references were divided into 15 clusters by CiteSpace software (Figure 5A), while keywords were divided into 6 clusters by VOSviewer (Figure 5C). Cluster numbers were sorted according to the size of the clusters, which indicates that larger clusters mean more relevant studies or keywords within them. In the keyword cluster #0, the common keywords such as arthritis, gouty arthritis, inflammation, monosodium urate, nlrp3 inflammasome, IL-1β, etc., indicated that this cluster mainly focused on the study of molecular mechanism and signal pathway in gouty arthritis (21, 34, 35). In the keyword cluster #1, the common keywords were gout, epidemiology, prevalence, population, care, quality of life, management, etc., which showed that this cluster mainly focused on the epidemiological and sociological research of gout (6, 7, 36). This hotspot received continuous attention from 2010 to 2017. The common keywords in keyword cluster #2 were hyperuricemia, risk factors, metabolic syndrome, cardiovascular disease, obesity, blood pressure, etc., indicating that this cluster concentrated mainly on the study of risk factors and complications of gout (37, 38). This hotspot received sustained attention from 2002 to 2010. In the keyword cluster #3, the common keywords were diagnosis, dual-energy CT, MRI, Ultrasound, Tophaceous, etc., which suggested that this cluster mainly focused on the research of gout diagnosis (39, 40). Common keywords such as allopurinol, febuxostat, trials, colchicine, efficacy, ULT, etc. in keyword cluster #4 demonstrated that this cluster focused mainly on therapeutic drug studies for gout prevention and gout flare (41, 42). This hotspot has been sustained since 2013. Finally, the common keywords in keyword cluster #5 were genome-wide association, gene, loci, mutations, excretion, kidney, transporter, etc., indicating that this cluster concentrated on the study of gout genetics and urate metabolism (43, 44).

The excretion of urate is regulated by the kidney and gut, and inadequate urate excretion increases serum urate concentrations. To date, urate excretion in the kidney is known to be collaborated by multiple transporters, however, transporters for gut urate excretion are poorly understood (3, 45). Urate concentration is the most important factor in the formation of MSU crystals, but low temperatures, physiological pH between 7 and 10, high concentrations of sodium ions, and components of synovium and cartilage also promote MSU crystallization (46, 47). MSU crystals could activate the NLRP3 inflammasome in macrophages and monocytes which is particularly associated with the initiation of gout flares (34, 48). Moreover, factors, such as free fatty acids, gut microbiota, and other microbiological components, can induce an acute inflammatory response in the presence of deposited MSU crystals (49, 50). Advanced gout is characterized by tophi, chronic gouty synovitis, and structural joint damage.

In the presence of typical signs and symptoms, the diagnosis of gout has a high level of certainty. Of note, hyperuricaemia, which affects more than 20% of men and 4% of women in the population, is not sufficient to diagnose gout in the absence of symptoms (2). In acute clinical situations, gout needs to be differentiated from musculoskeletal infections, rheumatoid arthritis and psoriatic arthritis. In cases of diagnostic uncertainty, microscopic confirmation of MSU crystals in either synovial fluid samples or material aspirated from tophi remains the gold standard for gout diagnosis (3, 26). Ultrasound and dual-energy CT can help diagnose gout when MSU crystals cannot be detected in the specimen. Ultrasonographic manifestations of gout include a double contour sign, intra-articular or intrabursal tophi, and a snowstorm appearance (40, 51). While, a positive dual-energy CT scan for gout is defined as the presence of typical color-coded MSU crystal deposits in the articular or periarticular areas, especially in the first metatarsophalangeal joints (39, 40).

Timeline plots of clusters can track the evolution of research hotspots and predict the research trends in the coming years (33). In addition, the burst detection algorithm developed by Kleinberg can capture the sharp increase in keyword popularity over a specific period, which can be used as an effective way to identify concepts or topics discussed actively during this period (52). Figures 5A,B revealed a shift in the research foci of gout in the order of clinical features, pathological mechanisms, complications, gouty arthritis, epidemiology, and dual-energy CT to drug clinical trials. In recent years, the research hotspot has perhaps been the development and experimentation of drugs for the prevention and treatment of gouty arthritis, which is predicted to receive continuous attention in the future as well. ULT is essential for the prevention of gout flares. The classical drugs are xanthine oxidase inhibitors (e.g. Allopurinol, Febuxostat) and uricosuric drugs (e.g. probenecid, sulfinpyrazone, benzbromarone, lesinurad) (3, 7). The effectiveness, adherence, usage timing, and safety of these drugs have been attracting a lot of attention (41, 42). Activation of the NLRP3 inflammasome by MSU crystals with the release of IL-1β is widely accepted as the major mechanism of gouty arthritis. Therefore, many drugs have been developed around NLRP3 inflammasome inhibitors and IL-1 inhibitors in recent years, such as celastrol (35), loganin (34), tetrahydropalmatine (48), anakinra (53), and dapansutrile (54). Besides, some new strategies for gout management have achieved satisfactory clinical results, such as storytelling intervention and nurse-led care, due to the low adherence to ULT (55, 56).

Lifestyle modifications such as weight loss, reduced intake of purine-rich foods, avoidance of alcohol and fructose-containing beverages, and increased consumption of dairy products may have an important role in the prevention of gout at the population level (22, 24, 31). Physical exercise and weight loss for overweight or obese patients with gout may have beneficial effects on serum urate levels (57). However, there is little evidence that dietary management alone can lead to durable or clinically meaningful reductions in serum urate concentrations once MSU crystal deposition has occurred and clinically evident gout is present (26, 58). Avoidance of specific foods that trigger gout flares may provide clinical benefit to gout patients, possibly by affecting pro-inflammatory pathways rather than altering serum urate levels (59). The effect of lifestyle modifications on gout urgently requires further high-quality clinical studies, such as prospective randomized controlled trials.

## Limitations

We have to acknowledge that this study has some inherent limitations in bibliometrics. First, the databases are constantly updated and only the WoSCC database was analyzed in this study. Some articles published in other databases may be omitted. However, as described in previous studies, WoSCC was the most commonly used and sufficiently large database for bibliometric analysis to reflect the current status in a given field (12, 15). Second, only English publications were selected and non-English publications were ignored, which implies that the value of non-English publications may be underestimated and would bias the study toward countries publishing in international journals with English as the main language. Third, this study did not perform a dual map overlay analysis which could reflect the relationship between journals and cited journals. Exploring connections among journals may help readers understand trends of publications and citations (11, 18). Finally, the impact of recently published high-quality articles may also be underestimated because they may not have sufficient time to accumulate enough citations. A follow-up study could be conducted in the future to evaluate the influence of these articles in the field.

## Conclusion

In summary, this study conducted a comprehensive bibliometric analysis of global publications on gout from 2001 to 2021. Our findings suggested that gout has attracted considerable and growing attention from scholars. Up to now, the USA and New Zealand have been leaders in this field, which is inseparable from adequate funding sources. University of Auckland and Professor Dalbeth Nicola were the most prolific institutions and influential authors, respectively. Rheumatology was the most prolific and influential journal in gout research. Gout research hotspots have shifted over time in the following order: clinical features, pathological mechanisms, complications, gouty arthritis, epidemiology, and dual-energy CT to drug clinical trials. The development and experimentation of drugs for the prevention and treatment of gouty arthritis are considered to be important research directions for the future, which would benefit from the combination of clinical epidemiological evidence and animal experiments. Overall, researchers, especially newcomers, could benefit from this bibliometric analysis as they could clearly understand the basic knowledge structure of the field, including countries, institutions, authors and journals, and be inspired by the research hotspots and frontiers.

## Data Availability Statement

The original contributions presented in the study are included in the article/Supplementary Material, further inquiries can be directed to the corresponding authors.

## Author Contributions

PW and PL: writing-original draft. PW and YZ: conceptualization, project administration, and writing-review and editing. PW, PL, and BZ: data curation and methodology. PW, BZ, and YZ: formal analysis, validation, visualization, and software. All authors contributed to the article and approved the submitted version.

## Funding

This work was supported by the Youth Cultivation Project of Xi'an Health Commission (Program No. 2020qn18) and the Key Research and Development Program of Shaanxi Province (Program No. 2022SF-237).

## Conflict of Interest

The authors declare that the research was conducted in the absence of any commercial or financial relationships that could be construed as a potential conflict of interest.

## Publisher's Note

All claims expressed in this article are solely those of the authors and do not necessarily represent those of their affiliated organizations, or those of the publisher, the editors and the reviewers. Any product that may be evaluated in this article, or claim that may be made by its manufacturer, is not guaranteed or endorsed by the publisher.
