# Supplementary material for: Mapping Knowledge Structure and Global Research Trends in Gout: A Bibliometric Analysis From 2001 to 2021
Source: Front Public Health. 2022 Jun 29;10:924676. doi: 10.3389/fpubh.2022.924676 (PMC9277182; doi:10.3389/fpubh.2022.924676)
Supplement: Supplementary file 1 [file Table_1.DOCX]

| **Supplementary Table 1** Correlation analysis between country-specific characteristics and bibliometric indices for gout research | | | |
| --- | --- | --- | --- |
| Country-specific characteristics | Bibliometric indices | R | P-value |
| Gross domestic product | Total publications | 0.826 | 0.000 |
|  | Total citations | 0.759 | 0.000 |
|  | Average citations | 0.385 | 0.000 |
| Gross domestic product per capita | Total publications | 0.525 | 0.000 |
|  | Total citations | 0.579 | 0.000 |
|  | Average citations | 0.464 | 0.000 |
| population | Total publications | 0.427 | 0.000 |
|  | Total citations | 0.289 | 0.007 |
|  | Average citations | -0.049 | 0.659 |
| International collaborations | Total publications | 0.865 | 0.000 |
|  | Total citations | 0.864 | 0.000 |
|  | Average citations | 0.585 | 0.000 |
